# Relationship between bone mineral density and alcohol intake: A nationwide health survey analysis of postmenopausal women

**DOI:** 10.1371/journal.pone.0180132

**Published:** 2017-06-29

**Authors:** Hae-Dong Jang, Jae-Young Hong, Kyungdo Han, Jae Chul Lee, Byung-Joon Shin, Sung-Woo Choi, Seung-Woo Suh, Jae-Hyuk Yang, Si-Young Park, Chungwon Bang

**Affiliations:** 1 Department of Orthopaedic Surgery, Soonchunhyang University Hospital, Seoul, South Korea; 2 Department of Orthopedics, Korea University Hospital, Ansan, South Korea; 3 Department of Biostatistics, College of Medicine, Catholic University, Seoul, South Korea; 4 Scoliosis Research Institute, Department of Orthopedics, Korea University Medical College, Guro Hospital, Seoul, South Korea; 5 Department of Orthopaedic Surgery, Korea University, College of Medicine, Anam Hospital, Seoul, South Korea; 6 Department of Orthopaedic Surgery, Cheonan Hospital, Soonchunhyang University, Chungcheongnam-do, South Korea; Oklahoma State University, UNITED STATES

## Abstract

**Objectives:**

Among a variety of relevant factors of osteoporosis, the association between alcohol intake and postmenopausal women’s bone mineral density (BMD) by using data from the Korean National Health and Nutrition Examination Survey was evaluated in this study.

**Materials and methods:**

Among a total of 31,596 subjects, males, premenopausal women, participants without BMD data were excluded. Finally, a total number of subjects in the study was 3,312. The frequency and amount of alcohol intake were determined by self-reported questionnaires, and BMD was measured by dual-energy x-ray absorptiometry.

**Results:**

Mean femoral BMD for light drinkers was statistically significantly greater than that for heavy drinkers and non-drinkers. We observed the characteristic trends for BMD by drinking frequency; the mean BMD gradually increased from non-drinkers to the participants who drank 2–3 times per week; these participants exhibited the highest BMD. Participants who drank alcohol greater than 4 times per week showed a lower BMD. In the risk factor analysis, the adjusted odds ratio for osteoporosis (at femoral neck) was 1.68 in non-drinkers and 1.70 in heavy drinkers compared with light drinkers.

**Conclusions:**

Light alcohol intake (2–3 times per week and 1–2 or 5–6 glasses per occasion) in South Korean postmenopausal women was related to high femoral BMD. Non-drinkers and heavy drinkers had approximately a 1.7-times greater risk for osteoporosis than light drinkers.

## Introduction

Osteoporosis (defined as bone mineral density (BMD) > 2.5 standard deviations below the young adult mean by World Health Organization (WHO)) is an enormous public health problem both in the medical and socioeconomic fields; presently, it affects more than 200 million people worldwide.[1] Osteoporosis is directly related to fragility fractures, and it is estimated that proportion of Asian people who suffered from hip fracture will increase to 37% of the people who succumb to hip fractures worldwide by 2025, and this number will increase to 45% by 2050.[2] Moreover, there are no perceived symptoms of osteoporosis, it is difficult to identify and, therefore, people do not begin treatment regimens early enough to prevent fractures and other related conditions. Thus, one of the most active means of management is to prevent the development of the disease by assessing risk factors.

Osteoporosis has multiple causes such as genetic and environmental factors including diet pattern.[1, 3–9] Among the dietary factors, alcohol intake is known to have a variety of influences on the bone health.[10–17] Based on the traditional consensus, chronic alcohol intake is known to thwart the development of ideal peak bone mass in young people and accelerate bone loss in elderly patients.[11, 13, 18, 19] However, several articles have identified positive effects of alcohol on bone density.[11, 14, 16, 17] These seemingly conflicting data have sparked recent controversies. In addition, the optimum amount and frequency of alcohol intake for the cited beneficial effects on bone health have not been clarified, and a number of studies have suggested contradictory effects of drinking according to age, gender, and menopausal state. For example, quantities of alcohol that appear to benefit postmenopausal (PMP) women seem to be disadvantageous for premenopausal women.[12, 15, 17] Also, heavy alcohol consumption has been associated with low BMD and high risk for hip fractures, whereas those in a moderate alcohol consumption group exhibited increased BMD. About these encountered features, previous reports suggested several hypotheses; a hormonal theory based on the possibility of transmutation of parathyroid hormone or calcitonin, phenolic theory based on its stimulation of estrogen receptors in osteoblast to increase BMD.[10, 12] Additional point to note is that South Korea leads Asia and ranked 15th in the world in terms of alcohol consumption according to the WHO’s 2014 report examined 196 countries.[20]

Despite these clinically important findings, to our knowledge, there is no population-based study that has examined the relationship between BMD and alcohol consumption in Korean PMP women. The aim of this study was to evaluate the association between alcohol intake and PMP women’s bone health by using data from the Korean National Health and Nutrition Examination Survey (KNHANES).

## Materials and methods

### Study population

**Fig 1:**
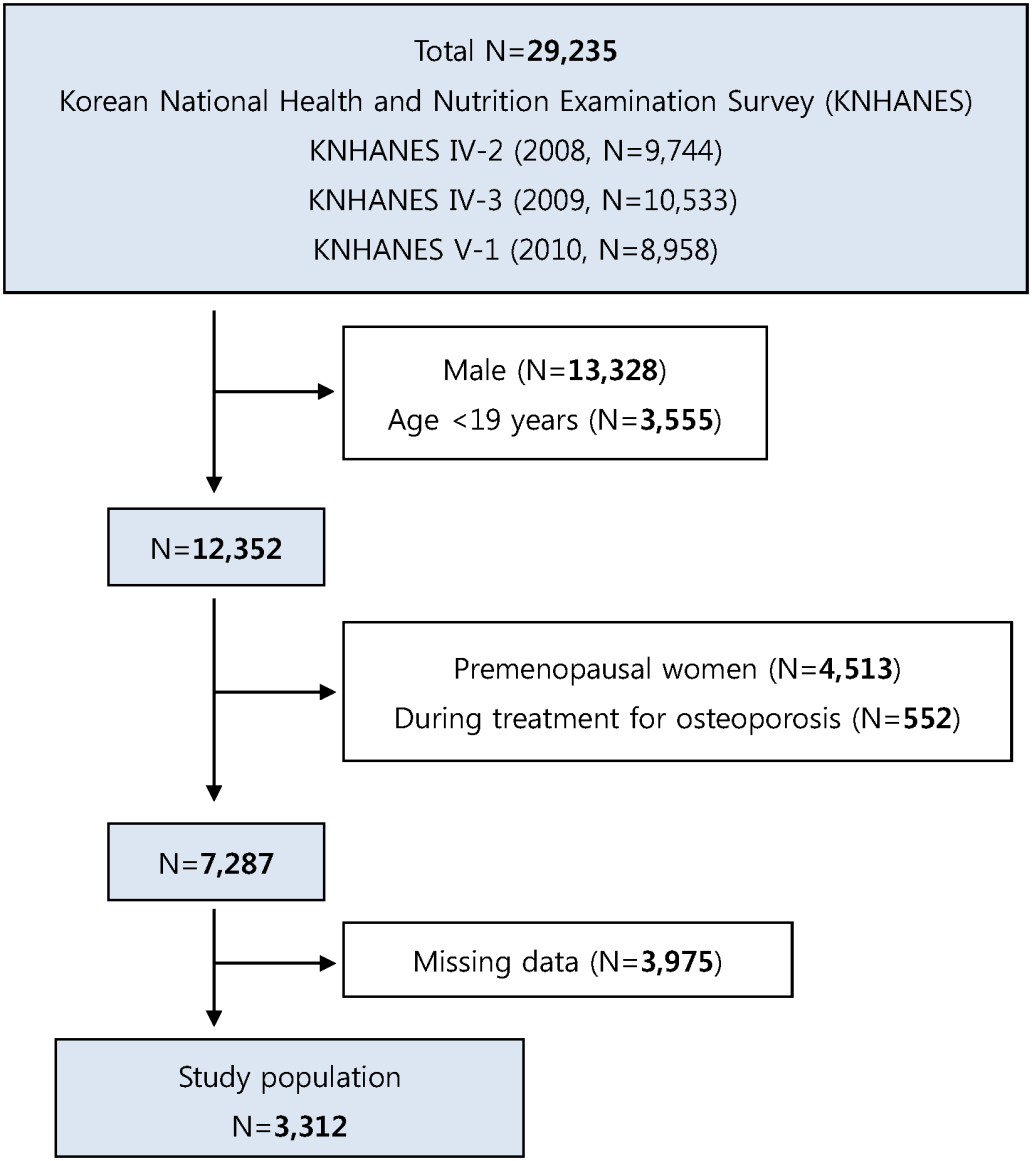
Flow chart of the inclusion and exclusion for subjects.

Data were obtained from the fourth KNHANES (KNHANES IV: 2008–2009) and the fifth KNHANES (KNHANES V: 2010) for this cross-sectional study. The KNHANES is an annual survey for nationwide health and nutrition from a representative sample performed by the Korea Centers for Disease Control and Prevention. The KNHANES comprised a health interview survey, a nutrition survey, and a health examination survey conducted by trained interviewers. This survey is a nationally representative study using a complex, stratified, multistage, clustered probability sample drawn from the South Korean population. Randomly selected sampling units were defined based on data from household registries, including geographic area, gender, and age groups. For the present study, we included only women with “natural” menopause, as shown in Fig 1. “Natural” menopause was defined as the absence of menses for 12 consecutive months in the absence of procedures that stopped menses including hysterectomy and oophorectomy. We excluded female participants without data for reproduction such as those who did not report a pregnancy history. All participants provided written informed consent, and the Institutional Review Board of the Korea Centers for Disease Control and Prevention approved the study protocol. (IRB No 2008-04EXP-01-C, 2009-01CON-03-2C, 2010-02CON-21-C)

### Measurement and classification of variables

Demographic variables including age, gender, amount and frequency of alcohol consumption, regular physical activity, marital status, smoking status, household income (4 groups by quartiles: low, medium to low, medium to high, and high), education level (low; <middle school, intermediate; high school, and high; >college), residential area, total energy intake, and various nutrient (calcium, energy, fat, and Vitamin D) intakes were collected using self-reported questionnaires. To assess the variables for reproduction, age at menarche, age at menopause, parity number, history of oral contraceptive use, history of hormone replacement therapy, and lactation history were evaluated. Parity was defined as the number of live births. Regular physical activity was defined as habitual physical activity that left the person slightly short-winded or that was tough compared to activities of daily living; if activity duration was greater than 30 minutes at a time and the frequency was greater than five times per week, the person was instructed to mark, “yes,” and if the person partook in physical activity less than that he or she was instructed to mark “no.” Body weight and height were measured, and body mass index (BMI) (kg/m^2^) was calculated. Waist circumference was measured at the narrowest point, between the lower costal margin and the iliac crest. An absolute BMD (g/cm^2^) of the lumbar spine (LS), total femur (TFM), femoral neck (NK), femoral trochanter (TR), and femoral intertrochanter (IT) were measured using dual energy x-ray absorptiometry (DEXA) with a DISCOVERY-W machine (Hologic, Inc., Bedford, MA, USA). According to the WHO criteria, osteoporosis was defined as a BMD T-score less than -2.5.

### Assessment of alcohol consumption

We assessed alcohol consumption by recording the frequency and amount of alcohol intake per occasion during the past 12 months with a self-reported questionnaire. Drinking frequency was divided into several groups by two different methods. In the first method, the three groups were: less than once per month (non-drinker), greater than once per month but less than twice per week (light drinker), and greater than twice per week (heavy drinker). In the second grouping method, participants were divided into seven groups: lifetime non-drinker, less than once per year, less than once per month, approximately one time per month, 2–4 times per month, 2–3 times per week, and greater than 4 times per week. In the present study, one standard glass was defined as roughly 8 g of pure alcohol, which is equal to what is contained in 220 mL of regular beer with approximately 4.5% alcohol and 50 ml of Korean distilled liquor (So-ju) containing approximately 19% alcohol. For drinking amount, the number of glasses participants drank per occasion was categorized into non-drinker, 1–2 glasses, 3–4 glasses, 5–6 glasses, 7–9 glasses, or greater than 10 glasses. Additionally, we defined high-risk drinkers as those who consumed alcohol more than twice per week and 5 glasses per occasion. Alcohol Use Disorders Identification Test (AUDIT) scores were also assessed; the AUDIT is a questionnaire based on alcohol drinking habits and behaviours related to alcohol consumption. Participants were divided into three groups by AUDIT score: less than 5, 5–9, and greater than 9.[21–25]

### Statistical analysis

Data are presented as a mean ± standard error (SE) or as percentages with SE. The SEs of percentage were the statistics that represented the precision for the estimate of categorical data in the KHANES and calculated by using the stratified, clustered, and weighted method to take into account the complex sampling design. In order to compare mean absolute BMD between the subgroups divided by amount and frequency of drinking, the degree of drinking risk, and AUDIT score, Students *t*-test, and analysis of covariance (ANCOVA) were used. To evaluate the association between alcohol consumption and osteoporosis, multivariable logistic regression analysis was used. In the adjusted analyses, age, BMI, smoke, exercise, education, income, calcium intake, energy intake, fat intake, and Vitamin D intake were used as confounding variables. Parameters with a P-value less than 0.15 for the *t*-tests and ANCOVA were selected for multivariable analyses. Statistical analysis was performed with SAS survey procedures (version 9.3; SAS Institute, Cary, NC, USA) in a manner that reflected sampling weights and provided nationally representative estimates. P-values less than 0.05 were considered statistically significant.

## Results

Among a total of 31,596 subjects, 25,534 (80.8%) participants were included in the health interview and examination survey. We excluded males, premenopausal women, and PMP women without BMD data. After ineligible subjects were excluded, the total number of subjects in the study was 3,312 (Fig 1). Demographic data and results for alcohol consumption and BMD are shown in Table 1.

**Table 1:** Demographic data and results of alcohol consumption and bone quality.

n	3312
Age (yr)	62.6 ± 0.2
BMI (kg/m^2^)	24.2 ± 0.1
Waist circumference (cm)	82.2 ± 0.3
**Socioeconomic and health status**	
High education	22.2% (1.1)
Low income	32.5% (1.2)
Regular physical activity	22.3% (1.0)
Smoke	4.6% (0.5)
DM	14.8% (0.8)
Calcium intake a day (mg)	426.7 ± 8.9
Energy intake a day (kcal)	1559.6 ± 16.7
Vitamin D status (serum 25-Hydroxyvitamin D, ng/mL)	17.0 ± 0.18
**Frequency of drinking**	
Non-	52.9% (1.2)
Light-	44.9% (1.2)
Heavy-	2.1% (0.3)
**Time(s) / period**	
Never	34.8% (1.1)
< 1 / yr	18.2% (0.8)
< 1 / mo	21.3% (0.9)
1 / mo	8.7% (0.6)
2 ~ 4 / mo	10.7% (0.6)
2 ~ 3 / wk	4.1% (0.4)
> 4 / wk	2.2% (0.3)
**AUDIT scores**	
< 5	89% (0.7)
5~9	7.2% (0.5)
> 9	3.8% (0.4)
**High-risk drinker**	4% (0.6)
**Amount of drinking**	
< 1	52.9% (1.2)
1 ~ 2	31.7% (1)
3 ~ 4	10.3% (0.7)
5 ~ 6	3% (0.4)
7 ~ 9	1.3% (0.2)
> 9	0.7% (0.2)
**BMD (g/cm**^**2**^**)**	
Lumbar spine	0.810 ± 0.003
Total femur	0.778 ± 0.003
Femoral neck	0.628 ± 0.002
Femoral trochanter	0.566 ± 0.002
Femoral intertrochanter	0.942 ± 0.003

Data are presented as mean ± SE or percentage (SE)

yr: year; BMI: body mass index; DM: diabetes mellitus; Non-: Non-drinker; Light-: Light drinker; Heavy-: Heavy drinker; mo: month; wk: week; AUDIT: Alcohol Use Disorders Identification Test; High-risk drinker: consumed alcohol greater than twice per week and 5 glasses per occasion; BMD: bone mineral density

### Mean BMD according to alcohol consumption

**Fig 2:**
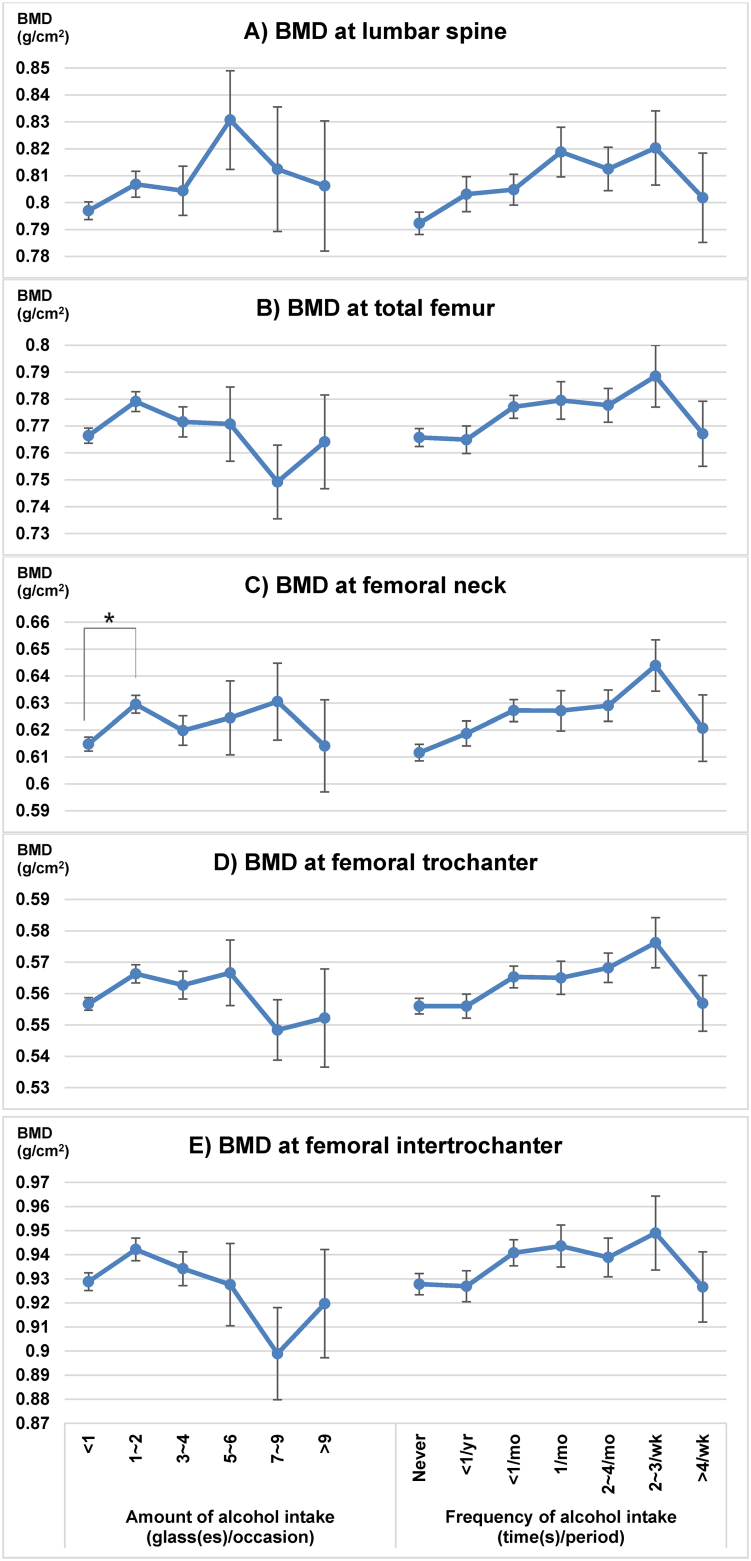
Mean BMD comparisons according to alcohol consumption. Relationship between alcohol consumption and (A) BMD at lumbar spine (B) BMD at total femur (C) BMD at femoral neck (D) BMD at femoral trochanter (E) BMD at femoral intertrochanter. Statistical significance * *p* <0.05. BMD: bone mineral density; yr: year; mo: month; wk: week.

Mean BMDs of light drinkers were greater than those of non-drinkers (at TFM, NK, TR, and IT) and heavy drinkers (at TR and IT) with statistically significant difference. Mean BMDs of participants with AUDIT scores 5–9 were equal to or greater than those of the other subgroups (AUDIT scores <5 and >9). Mean femoral BMDs of high-risk drinkers were less than those of the non-high-risk drinkers. However, there were no significant differences between BMDs of subgroup divided by AUDIT scores or whether the participants were high-risk drinkers or not (Table 2). The subgroup that showed the highest BMD based on drinking amount was 1–2 glasses per occasion at the TFM and IT, 5–6 glasses at the LS and TR, and 7–9 glasses at the NK. Fig 2 presents the characteristic trends for BMD at all sites; the mean BMD of subgroups by drinking frequency gradually increased from non-drinkers to the participants who drank 2–3 times per week; these participants exhibited the highest BMD. With further increased alcohol consumption, steep decreasing trends were observed. Participants who drank alcohol greater than 4 times per week showed a lower BMD. However, there were no significantly different analyses and subgroups, except higher femoral neck BMD (0.633 ± 0.004 g/cm^2^) of 1 or 2 glasses/occasion group compared to BMD of no intake group (0.619 ± 0.004 g/cm^2^).

**Table 2:** Mean BMD comparisons according to the alcohol consumption.

	BMD (g/cm^2^)				
	Total Femur	Femoral neck	Femoral trochanter	Femoral inter-trochanter	Lumbar spine
**Frequency of drinking**^†^					
Non-	0.766 (0.005)	0.615 (0.005)	0.557 (0.004)	0.929 (0.007)	0.797 (0.007)
Light-	0.777 (0.006)	0.628 (0.006)	0.566 (0.004)	0.94 (0.007)	0.808 (0.008)
Heavy-	0.749 (0.016)	0.617 (0.016)	0.543 (0.011)	0.902 (0.02)	0.812 (0.023)
*p*	0.009**^‡^	0.0081**^‡^	0.0036**^§^	0.0235*^§^	0.1069
**AUDIT scores**^†^					
< 5	0.776 (0.004)	0.625 (0.004)	0.565 (0.003)	0.940 (0.005)	0.806 (0.006)
5–9	0.779 (0.008)	0.631 (0.008)	0.565 (0.006)	0.943 (0.010)	0.819 (0.01)
> 9	0.757 (0.012)	0.610 (0.01)	0.551 (0.008)	0.916 (0.015)	0.794 (0.014)
*p*	0.2007	0.2328	0.2251	0.2401	0.3032
**High-risk drinkers**					
Non-high-risk	0.799 (0.003)	0.648 (0.003)	0.582 (0.002)	0.966 (0.004)	0.826 (0.004)
High-risk	0.766 (0.019)	0.638 (0.018)	0.559 (0.012)	0.920 (0.024)	0.834 (0.026)
*p*	0.0941	0.5863	0.0773	0.0598	0.7617

Statistical significance *p<0.05, **p<0.01.; (): standard error

^†^: Comparative analysis using analysis of covariance (ANCOVA)

Adjusted ANCOVA test: age, BMI, smoke, exercise, education, income, calcium intake, energy intake, fat intake, and Vitamin D intake

Post hoc test results

^‡^: Light drinker’s BMD was significantly greater than non-drinker’s BMD

^§^: Light drinker’s BMD was significantly greater than both BMD of non- and heavy drinker

BMD: bone mineral density; Non-: Non-drinker; Light-: Light drinker; Heavy-: Heavy drinker; AUDIT: Alcohol Use Disorders Identification Test

### Association between osteoporosis and alcohol consumption

The results of multivariable logistic regression analysis with adjustment for age, BMI, smoking, exercise, education, and income showed no significant association between the alcohol consumption and osteoporosis at every BMD measurement sites except the femoral neck. The adjusted odds ratio (OR) for osteoporosis (at the FN) was 1.63 in non-drinkers and 1.76 in heavy drinkers compared to light drinkers (Table 3). The adjusted OR for osteoporosis (at any site) was 1.24 in non-drinkers and 1.08 in heavy drinkers compared with light drinkers. Consequently, non-drinkers showed significantly higher risk for osteoporosis compared to light drinkers.

**Table 3:** Calculated risks of osteoporosis in the Korean postmenopausal women according to the alcohol consumption.

	Osteoporosis*		Osteoporosis^†^	
Frequency of drinking	Adjusted OR^‡^	Adjusted OR^§^	Adjusted OR^‡^	Adjusted OR^§^
**Light- (Reference)**	1		1	
**Non-**	1.63 (1.26–2.1)	1.63 (1.23–2.12)	1.25 (1.02–1.53)	1.24 (1.01–1.53)
**Heavy-**	1.72 (0.52–5.7)	1.76 (0.54–5.68)	1.04 (0.45–2.37)	1.08 (0.49–2.40)

(): 95% confidence interval

*Osteoporosis: BMD T-score < -2.5 at femoral neck

^†^Osteoporosis: BMD T-score < -2.5 at any site

^‡^Adjusted: age, BMI

^§^Adjusted: age, BMI, smoke, exercise, education, income

OR: Odds ratio; Light-: Light drinker; Non-: Non-drinker; Heavy-: Heavy drinker

## Discussion

The present study showed the results that the mean femoral BMD of light drinkers was greater than those of non-drinkers and heavy drinkers (at TR and IT) with statistically significant difference. Subgroup analysis showed higher BMD of participants with AUDIT scores 5–9 compared to the other score groups (<5 and >9), and less femoral BMD of high-risk drinkers compared to non-high-risk drinkers. However, there were no statistically significant differences by AUDIT score and high-risk drinking. More specifically, participants who drank alcohol 2–3 times per week and 1–2 or 5–6 glasses per occasion showed a higher BMD compared to all other groups. In a risk factor analysis of PMP women’s osteoporosis, non-drinkers exhibited a higher risk factor than heavy drinkers and light drinkers. Based on the evidence from both lower and upper 95% confidence intervals (CI), non-drinkers showed the greatest risk for osteoporosis (OR: 1.68, 95% CI: 1.31–2.18). In South Korea, a few cross-sectional studies using KNHANES data investigated the effects of alcohol on bone health.[26, 27] In a study in healthy adult men, all the relationships between alcohol intake and BMD faded into insignificance after multivariable adjusted analyses. Conversely, in young Korean women, frequent and copious drinking was related to low BMD and osteoporosis, especially in the femoral neck. These contradictory effects likely stem from a variety of complex factors of participants including age, gender, hormonal changes, and related socioeconomic environments.

The present study has a few limitations. First, detailed analysis regarding participant previous medical treatment for osteoporosis was not included due to the limits of the dataset. The second limitation was the exclusion of postsurgical menopause participants. Because gynecological health screening and related surgery have increased in this ageing society, low BMD and its risk factors in iatrogenically induced menopausal women should be more thoroughly evaluated in the future. Despite these limitations, the present study suggests that osteoporosis and fragility fractures in PMP women can be reduced by controlling alcohol intake.

Our result that non-alcohol drinkers and heavy drinkers showed lower BMDs and a higher risk of osteoporosis in the PMP women compared to light drinkers should be interpreted cautiously. Due to the complex relationship between alcohol consumption and BMD highlighted in the present study, multidisciplinary assessment for other related socioeconomic conditions is essential to evaluate and promote individual women’s bone health.

## Conclusions

In the present study, light alcohol intake (2–3 times per week and 1–2 or 5–6 glasses per occasion) in South Korean postmenopausal women was related to high femoral BMD (total femur, femoral neck, femoral trochanter, and femoral intertrochanter). Non-drinkers and heavy drinkers had approximately a 1.7-times greater risk for osteoporosis than light drinkers.
